# Severe heat stroke complicated by multiple cerebral infarctions: a case report

**DOI:** 10.1186/s13256-020-02596-2

**Published:** 2021-01-28

**Authors:** Ryo Kamidani, Hideshi Okada, Yuichiro Kitagawa, Keigo Kusuzawa, Masahiro Ichihashi, Yoshinori Kakino, Hideaki Oiwa, Ryu Yasuda, Tetsuya Fukuta, Naomasa Yoshiyama, Takahito Miyake, Haruka Okamoto, Kodai Suzuki, Noriaki Yamada, Tomoaki Doi, Takahiro Yoshida, Hiroaki Ushikoshi, Keisuke Kumada, Shozo Yoshida, Shinji Ogura

**Affiliations:** Advanced Critical Care Center, Gifu University Hospital, 1-1 Yanagido, Gifu, 501-1194 Japan

**Keywords:** Heat stroke, Intracranial hemorrhaging, Magnetic resonance angiography, Multiple cerebral infarctions

## Abstract

**Background:**

Heat-related illnesses include symptoms such as heat syncope/cramps, heat exhaustion, and life-threatening heat stroke. Usually, a heat stroke causes cerebellar ataxia, cognitive impairment, dysphagia, and aphasia. We report a very rare case of a patient who developed severe heat stroke complicated by multiple cerebral infarctions.

**Case presentation:**

An 80-year-old Asian woman was found lying unconscious at her house, with no air conditioner and closed windows; the highest outside temperature was 36.1 °C. She was brought to our hospital unconscious with a high bladder temperature (42.5 °C) and disseminated intravascular coagulation (DIC score 4). She was diagnosed with severe heat stroke and managed with rapid cooling, intravenous fluids therapy, antibiotic therapy, and anti-coagulation therapy for DIC. Anti-coagulation therapy consisted of treatment with recombinant thrombomodulin for 4 days (days 1–4) and recombinant antithrombin for 1 day (day 1). A head computed tomography (CT) and magnetic resonance imaging (MRI) examination were performed on day 3, because she was still unconscious. Diffuse-weighted imaging showed high-signal intensities, indicating multiple lesions. An intracranial magnetic resonance angiography showed normal results. Imaging indicated new multiple cerebellar infarctions complicated with DIC. A tracheotomy was performed on day 9 because her conscious condition had not improved. She was transferred to another hospital for subacute care on day 23.

**Conclusions:**

Early management of heat stroke using anti-DIC, anti-bacterial, and fluid resuscitation therapy can help prevent complications such as intracranial hemorrhaging.

## Background

Heat-related illnesses include diverse symptoms such as heat syncope/cramps, heat exhaustion, and life-threatening heat stroke [1]. Acute severe heat stroke may be associated with rhabdomyolysis, disseminated intravascular coagulation (DIC), acute renal failure, liver damage, acute respiratory distress disease syndrome, electrolyte imbalance, and neurologic complications [2–6]. The typical neurologic complications are cerebellar ataxia, cognitive impairment, dysphagia, and aphasia. We report a very rare case of a patient who developed severe heat stroke complicated by multiple cerebral infarctions.

## Case presentation

An 80-year-old Asian woman with Alzheimer dementia was found lying unconscious at her house, which had no air conditioner and the windows were kept closed; the highest outside temperature was 36.1 °C. There was no history of seizure, previous use of medication, diabetes mellitus, hypertension, alcohol abuse, smoking, or cardiac disease. During transportation, a physician began to assist her ventilation, and she was intubated because her SpO_2_ level was 78% under room air. She was brought by the ambulance with a physician onboard to our hospital unconscious. Her Glasgow Coma Scale score was 6 (eye, 1; verbal, 1; motor, 4), with a high bladder temperature (42.5 °C). On arrival, her blood pressure was 104/79 mmHg and pulse rate was abnormal at 110 beats/min. She was vomiting but had no traumatic scars. Results of an arterial blood gas examination are shown in Table 1. Laboratory data revealed renal dysfunction and an elevated white blood cell count at 13,890/μL (normal range 3000–9000/μL) (Table 1). Her DIC score was 5 points as per the DIC diagnostic criteria established by the Japanese Association for Acute Medicine (JAAM) on admission. On day 2, she met the criteria (5 points) of a different diagnostic system established by the International Society on Thrombosis and Hemostasis (ISTH) [7]. Her blood culture was sterile. An electrocardiogram, chest X-ray, and two-dimensional transthoracic echocardiography showed normal results. Serology laboratory tests for venereal disease, human immunodeficiency virus, and viral hepatitis markers (hepatitis A virus (HAV), hepatitis B virus (HBV), and hepatitis C virus (HCV)) were negative. No abnormal lesion was found on the head computed tomography (CT) examination performed on arrival (Fig. 2 upper panels).

**Table 1:** Laboratory findings at the time of admission

Complete blood cell counts	(Normal range)	Biochemistry	(Normal range)
White blood cells	13.2×10^9^ (3.5–9.1×10^9^) cells/L	Total protein	6.1 (6.7–8.3) g/dL
Red blood cells	4.0×10^12^ (3.7–5.0×10^12^) cells/L	Albumin	3.5 (3.8–5.2) g/dL
Hemoglobin	119 (113–152) g/L	Aspartate transaminase	44 (10–40) IU/L
Platelet	42×10^9^ (130–369×10^9^) cells/L	Alanine transaminase	15 (5–40) IU/L
Coagulation status		Lactate dehydrogenase	353 (115–245) IU/L
Activated partial thromboplastin time	40.8 (24.3–36.0) sec	Alkaline phosphatase	211 (115–359) IU/L
Prothrombin time	15.8 (10.5–13.5) sec	Creatinine	98.1 (41.5–69.8) μmol/L
Prothrombin time-international normalized ratio	1.42 (0.85–1.15)	Blood urea nitrogen	6.8 (2.8–7.8) mmol/L
Fibrinogen	2.8 (1.5–4.0) g/L	Total bilirubin	18.8 (5.1–20.5) μmol/L
Fibrin degradation product	70.5 (≤ 4.0) mg/L	Sodium	134 (136–147) mmol/L
D-dimer	42,900 (< 1000) μg/L	Potassium	3.6 (3.6–5.0) mmol/L
Arterial blood gas		Chloride	101 (98–109) mmol/L
F_I_O_2_	0.4	C-reactive protein	500 (≤ 3000) μg/L
pH	7.452 (7.35–7.45)	Blood sugar	10.4 (3.8–6.0) mmol/L
PaCO_2_	28.4 (32–45) mmHg	Haemoglobin A1c	36.6 (26.7–44.2) mmol/mol
PaO_2_	214 (69–116) mmHg		
HCO^3−^	19.6 (20–26) mmol/L		
Base excess	− 3 (− 3.3–+ 1.2) mmol/L		
Lactate	2.8 (0.5–1.2) mmol/L		

**Fig. 1:**
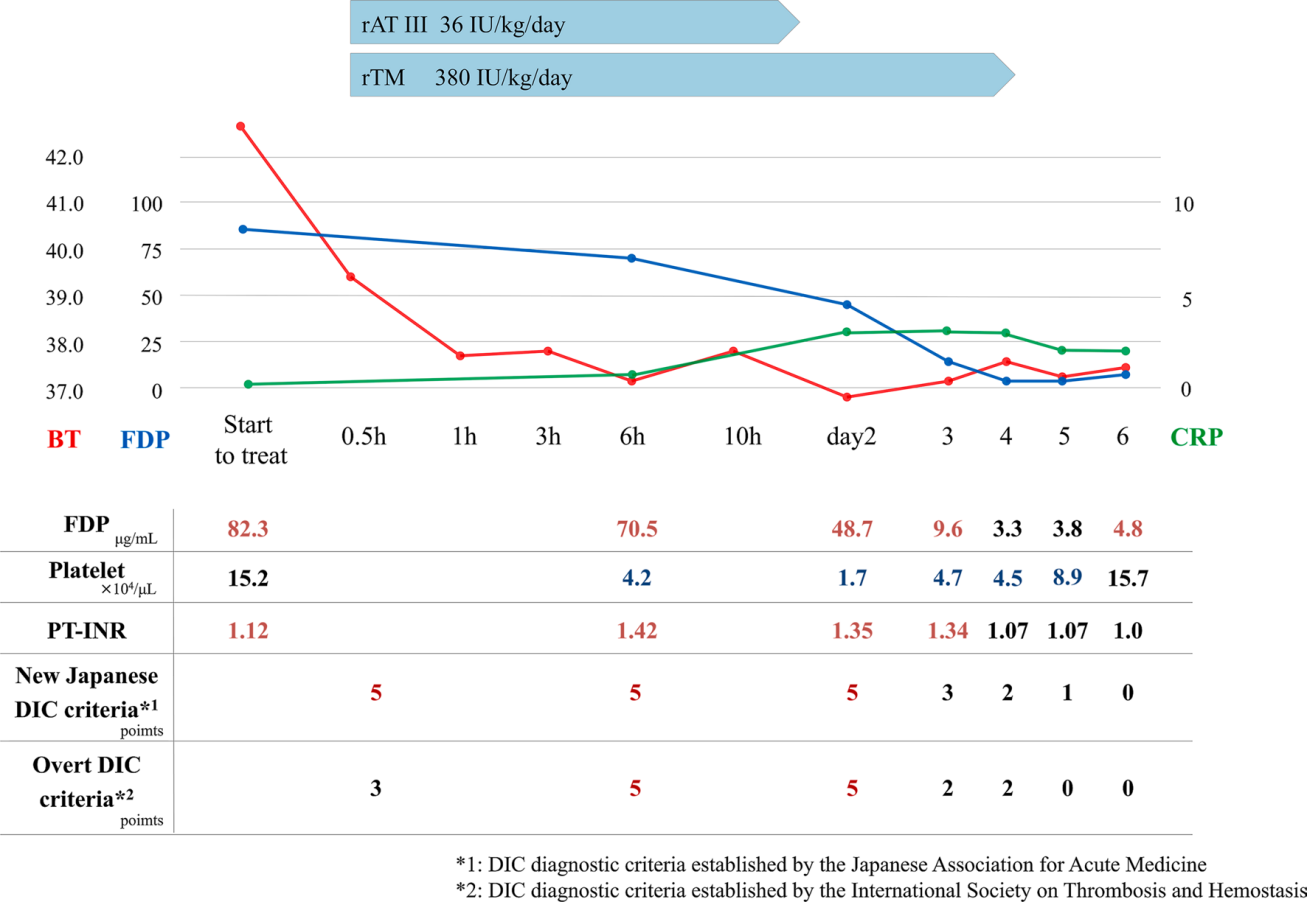
Summary of the clinical course. rAT: recombinant anti-thrombin, rTM: recombinant thrombomodulin, *BT* body temperature, *FDP* fibrin/fibrinogen degradation products, *PT-INR* prothrombin international normalized ratio

The patient was diagnosed with severe heat stroke, placed under intensive care, and managed with rapid cooling, intravenous fluid therapy, antibiotic therapy, and anti-coagulation therapy for DIC (Fig. 1). Anti-coagulation therapy consisted of treatment with recombinant thrombomodulin for 4 days (days 1–4) and recombinant antithrombin for 1 day (day 1). We transfused 10 U of platelet concentrate because her platelet count had decreased to 1.7×10^4^/μL due to exhaustion on day 2.

**Fig. 2:**
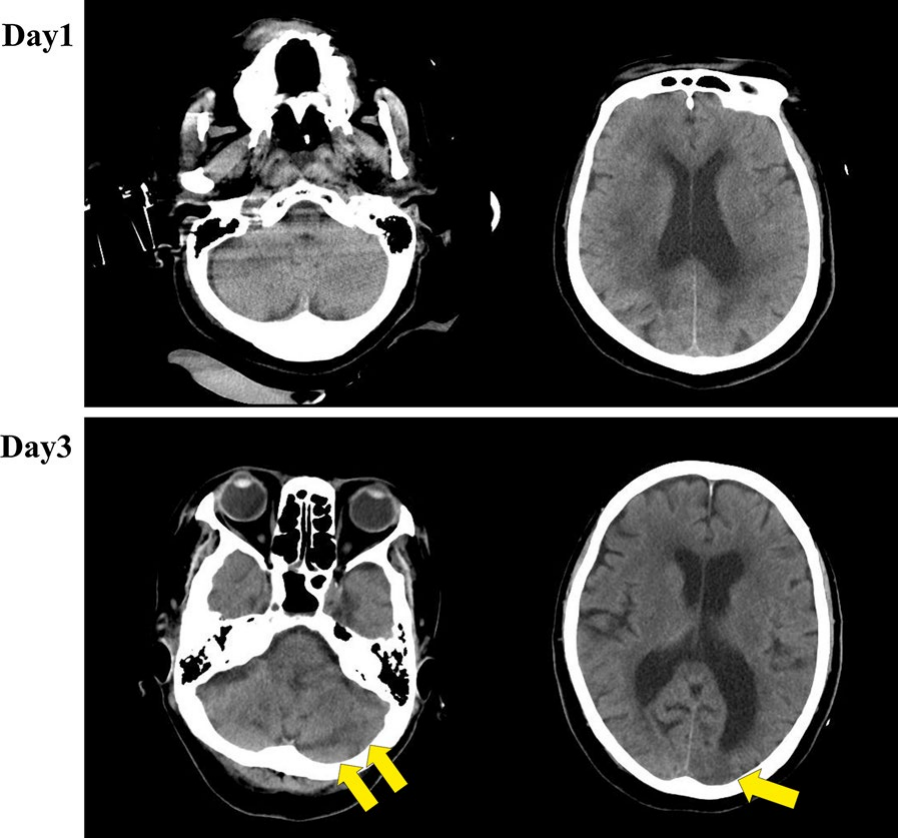
Head computed tomography on days 1 and 3. Head computed tomography (CT) examination on day 1 (upper panels) and day 3 (lower panels). No abnormal lesion is found on day 1. On day 3, low-intensity areas are detected in the cerebellum and left occipital lobe (yellow arrows).

**Fig. 3:**
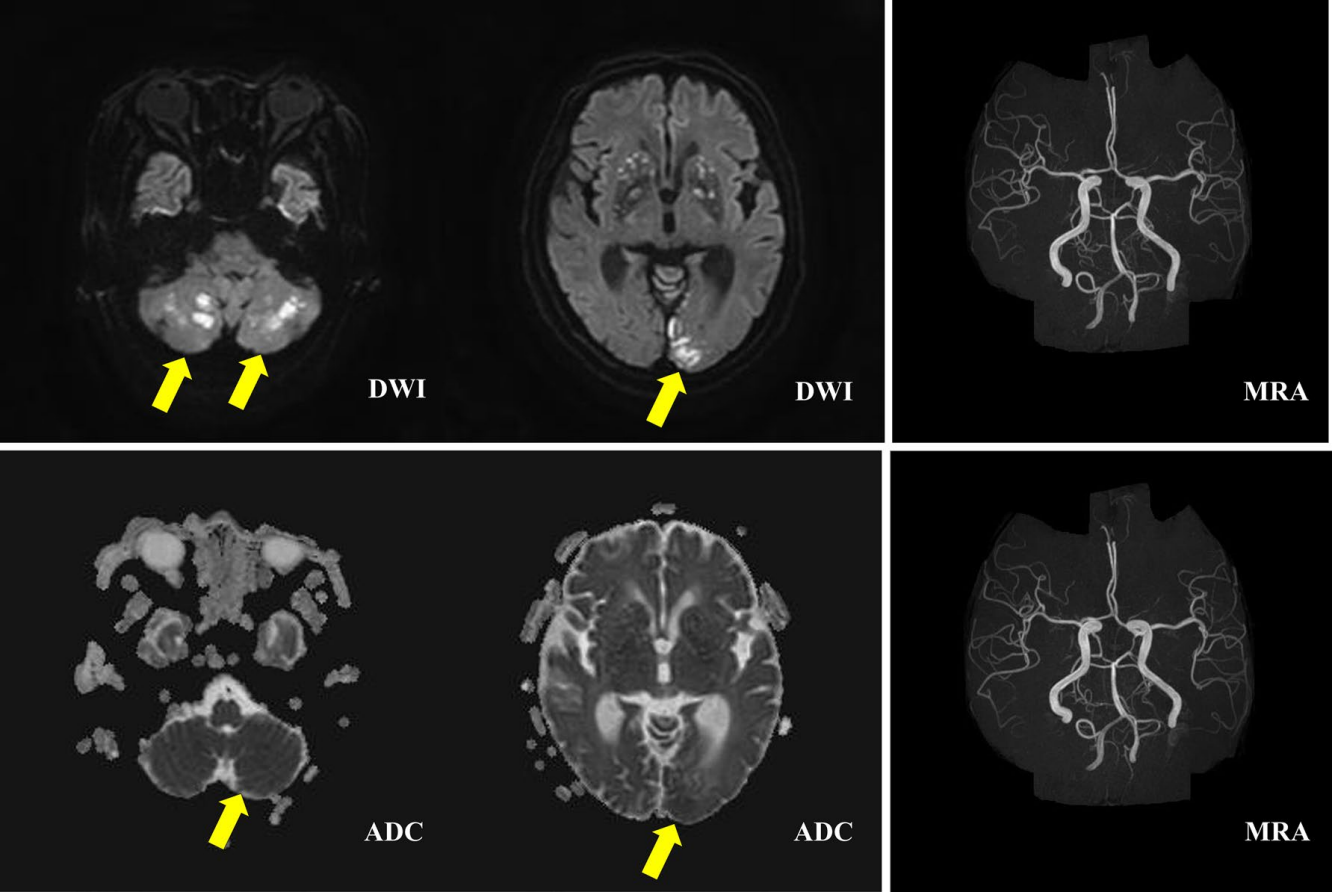
Brain magnetic resonance imaging on day 3. Diffuse-weighted imaging showing high-signal areas on the bilateral cerebellar hemisphere, occipital lobe, semioval center, actinic crown, and basal ganglia. The apparent diffusion coefficient (ADC) map shows a partial decline of the ADC value. An intracranial magnetic resonance angiography shows normal results. Yellow arrows indicated new multiple cerebellar infarctions.

Head CT (Fig. 2 lower panels**)** and magnetic resonance imaging (MRI) (Fig. 3) examinations were performed on day 3 because she was still unconscious. Diffuse-weighted imaging showed high-signal intensities in the bilateral cerebellar hemisphere, bilateral occipital lobe, and basal ganglia. Intracranial magnetic resonance angiography showed normal results. Imaging indicated new multiple cerebellar infarctions (Fig. 3). As described above, she had no arrhythmia or organic cardiac disease, and the location of the infarcts included the cerebellum. It was thought that heat stroke with DIC complicated the acute infarctions. A tracheotomy was performed on day 9 because her unconscious condition had not improved. She was transferred to another hospital for subacute care on day 23.

## Discussion and conclusions

Heat stroke is a serious and life-threatening emergency, with a high mortality rate (20%) [1, 8]. This is thought to be because heat stroke has several complications, especially neurological ones such as cerebellar ataxia, cognitive impairment, dysphagia, aphasia, other minor symptoms (irritability, irrational behavior, hallucinations, downbeat nystagmus, and opsoclonus), and severe features such as cranial nerve abnormalities, seizures, and coma [9]. It was previously reported that the incidence of after-effects from heat stroke on the central nervous system (CNS) is 1.5 % (22/1,441 cases) [10].

It is presumed that two factors caused the neurologic complications. One reason is that the CNS is highly sensitive to hyperthermia, especially the cerebellum, basal ganglia, anterior horn cells, and peripheral nerves. Bazille *et al.* and Malamud et al reported that Purkinje cells were highly sensitive to hyperthermia [3, 11]. A second reason is vasogenic edema and cytotoxic edema due to vascular hyperpermeability, which can be induced by hypercytokinemia. Hypercytokinemia may also cause destruction of the blood–brain barrier. Its radiological characteristic is posterior reversible encephalopathy syndrome-like because of restricted diffusion caused by edema.

Leakage of endotoxin due to bacterial translocation and cytokine release from muscles can activate white blood cells and the vascular endothelium, causing inflammation. This results in the release of inflammatory cytokines (for example, tumor necrosis factor α, interleukin-1β, and interferon-γ) and anti-inflammatory cytokines for example, interleukin-6 and interleukin-10), activation of coagulation (with reduced levels of proteins C and S, and antithrombin), and the inhibition of fibrinolysis [12].

Previous reports indicated that the dual effects of elevated PAI-1 activity and decreased t-PA activity in the fibrinolytic balance may be a major contributor to the pro-thrombotic shift of heat stroke, rather than the platelet-related pro-thrombotic activity, in human umbilical vein endothelium cells [13, 14]. Although these reports are in vitro studies, they indicate that endothelium cells exposed to hyperthermia are involved in fibrinolytic balance, as seen in clinical practice.

As described above, the inflammatory and coagulation response to heat stroke results in vascular endothelium disorder, microthrombosis, and DIC via strong inhibition of fibrinolysis, with subsequent improvement of hyperthermia. Clinical and laboratory diagnostic criteria and a scoring system for DIC have been established by the ISTH and JAAM (Tables 2 and 3). In Japan, we often use the JAAM diagnostic criteria (new Japanese criteria) for early diagnosis of DIC in the field of emergency and critical care medicine. Although the ISTH established two sets of criteria for overt and non-overt DIC, the non-overt DIC criteria are more appropriate for early diagnosis. A recent study showed that the diagnostic sensitivity of the new Japanese criteria was as high as that of the non-overt DIC criteria [15]. Furthermore, the new Japanese criteria allowed for the earliest diagnosis and the most accurate outcome prediction among all DIC criteria. In this case, the patient met the new Japanese criteria (5 points), but not the overt DIC criteria (3 points) on admission. However, she ultimately progressed to meeting the overt DIC criteria (5 points) on day 2. That is, we were able to institute early management of her DIC, but could not prevent the complications associated with DIC and hypercytokinemia.

**Table 2:** The DIC diagnostic criteria established by the Japanese Association for Acute Medicine (new Japanese Criteria)

	Score
SIRS score	
≥ 3	1
≤ 2	0
Platelet count (10^9^/L)	
< 80 or 50% decrease within 24 hours	3
< 120, 80 or 30% decrease within 24 hours	1
≥ 120	0
≥ 1.2	1
< 1.2	0
Fibrinogen level (g/L)	
< 3.5	1
≥ 3.5	0
Fibrin/fibrinogen degradation products (mg/L)	
≥ 25	3
≥ 10, < 25	1
< 10	0

If the total score was ≥ 4, DIC was diagnosed

*DIC* disseminated intravascular coagulation

**Table 3:** The DIC diagnostic criteria established by the International Society on Thrombosis and Hemostasis (overt DIC Criteria)

	Score
Platelet count (10^9^/L)	
< 50	2
< 100, ≥ 50	1
≥ 100	0
Elevated fibrin-related marker	
Strong increase	3
Moderate increase	2
No increase	0
Prolonged prothrombin time (seconds)	
≥ 6	2
< 6, ≥ 3	1
< 3	0
Fibrinogen level (g/L)	
< 1	1
≥ 1	0

If the total score was ≥ 5, overt DIC was diagnosed

*DIC* disseminated intravascular coagulation

In addition, increased intracranial pressure and autonomic dysfunction, caused by vasogenic and cytotoxic edema due to hypercytokinemia, leads to cerebral hypoperfusion and ischemia. In the present case, the patient had multiple infarctions in the bilateral cerebellar hemisphere, bilateral occipital lobe, and basal ganglia. DIC and hypercytokinemia induced by heat stroke cause microthrombosis, which results in small vessel ischemic damage and cerebral infarction. We hypothesized that these mechanisms were initiated by the infarctions because she had no history of cardiac disease or risk factors for vascular diseases.

There are few reports about CNS complications due to heat stroke, and acute infarction is especially rare. Our patient may be the first reported case of multiple cerebral infarctions due to heat stroke that had persistent neurologic features in the form of a coma.

In conclusion, early management of heat stroke using DIC therapy, anti-bacterial therapy, and fluid resuscitation therapy is required. Even if there is no DIC, anti-coagulant therapy is desirable considering the possible risk of an intracranial hemorrhage.

## Data Availability

The datasets obtained and analyzed in the current study are available from the corresponding author on reasonable request.

## Abbreviations

ADC: Apparent diffusion coefficient
CNS: Central nervous system
DIC: Disseminated intravascular coagulation
ISTH: International Society on Thrombosis and Hemostasis
JAAM: Japanese Association for Acute Medicine

## References

[1] Leon LR, Bouchama A. Heat stroke. Compr Physiol. 2015;5(2):611–47. 10.1002/cphy.c140017 25880507

[2] Adhami F, Liao G, Morozov YM, Schloemer A, Schmithorst VJ, Lorenz JN, et al. Cerebral ischemia-hypoxia induces intravascular coagulation and autophagy. Am J Pathol. 2006;169(2):566–83. 10.2353/ajpath.2006.051066 PMC1780162 16877357

[3] Bazille C, Megarbane B, Bensimhon D, Lavergne-Slove A, Baglin AC, Loirat P, et al. Brain damage after heat stroke. J Neuropathol Exp Neurol. 2005;64(11):970–5. 10.1097/01.jnen.0000186924.88333.0d 16254491

[4] de Galan BE, Hoekstra JB. Extremely elevated body temperature: case report and review of classical heat stroke. Neth J Med. 1995;47(6):281–7. 10.1016/0300-2977(95)00040-2 8569934

[5] Khosla R, Guntupalli KK. Heat-related illnesses. Crit Care Clin. 1999;15(2):251–63. 10.1016/s0749-0704(05)70053-1 10331127

[6] Romero JJ, Clement PF, Belden C. Neuropsychological sequelae of heat stroke: report of three cases and discussion. Mil Med. 2000;165(6):500–3. 10870374

[7] Taylor FB Jr., Toh CH, Hoots WK, Wada H, Levi M, Scientific Subcommittee on Disseminated Intravascular Coagulation of the International Society on T, et al. Towards definition, clinical and laboratory criteria, and a scoring system for disseminated intravascular coagulation. Thromb Haemost. 2001;86(5):1327–30. 11816725

[8] Leon LR, Helwig BG. Heat stroke: role of the systemic inflammatory response. J Appl Physiol (1985). 2010;109(6):1980–8. 10.1152/japplphysiol.00301.2010 20522730

[9] Jain RS, Agrawal R, Kumar S, Gupta PK. Stroke after piercing barbed wire injury: a time for introspection. J Stroke Cerebrovasc Dis. 2015;24(4):e97–100. 10.1016/j.jstrokecerebrovasdis.2014.11.029 25638052

[10] Nakamura S. Sequelae secondary to heat-related illness. Nihon Rinsho. 2012;70(6):969–74. 22690602

[11] Malamud N, Haymaker W, Custer RP. Heat stroke; a clinico-pathologic study of 125 fatal cases. Mil Surg. 1946;99(5):397–449. 20276794

[12] Bouchama A, Knochel JP. Heat stroke. N Engl J Med. 2002;346(25):1978–88. 10.1056/NEJMra011089 12075060

[13] Ang C, Dawes J. The effects of hyperthermia on human endothelial monolayers: modulation of thrombotic potential and permeability. Blood Coagul Fibrinolysis. 1994;5(2):193–9. 10.1097/00001721-199404000-00006 8054450

[14] Wojta J, Holzer M, Hufnagl P, Christ G, Hoover RL, Binder BR. Hyperthermia stimulates plasminogen activator inhibitor type 1 expression in human umbilical vein endothelial cells in vitro. Am J Pathol. 1991;139(4):911–9. PMC1886316 1928306

[15] Hayakawa M, Gando S, Hoshino H. A Prospective comparison of new Japanese criteria for disseminated intravascular coagulation: new Japanese criteria versus ISTH criteria. Clin Appl Thromb Hemost. 2007;13(2):172–81. 10.1177/1076029606299077 17456627

